# On the Role of Teacher–Student Rapport and Teacher Support as Predictors of Chinese EFL Students’ Affective Learning

**DOI:** 10.3389/fpsyg.2022.856430

**Published:** 2022-03-10

**Authors:** Yuchao Sun, Wenshu Shi

**Affiliations:** ^1^School of Foreign Studies, China University of Petroleum (East China), Qingdao, China; ^2^College of Foreign Languages, Qingdao Binhai University, Qingdao, China

**Keywords:** student affective learning, teacher–student rapport, Chinese EFL students, teacher support, structural equation modeling

## Abstract

Students’ affective learning is critical for their academic success; therefore, considerable attention has been devoted to the role of various student-related and teacher-related factors as predictors of student affective learning. Notwithstanding, the impact of two important teacher-related factors, namely teacher–student rapport and teacher support, has not been adequately researched. To address this gap, the present study sought to explore the role of teacher support and teacher–student rapport in Chinese English as a foreign language (EFL) students’ affective learning. To do so, three valid inventories of the variables were administered to 497 Chinese EFL students. Performing correlational analyses, favorable associations were found between teacher–student rapport, teacher support, and student affective learning. The predictive power of teacher support and teacher–student rapport was assessed using structural equation modeling (SEM). Chinese EFL students’ affective learning was shown to be largely influenced by teacher–student rapport and teacher support. The pedagogical implications and future directions are also discussed.

## Introduction

Students’ academic success is the principal concern of instructors in any educational setting, including English language classes (Karatas et al., 2015). To lead students toward success, instructors should not merely focus on the behavioral and cognitive domains of students’ learning. It means that the affective domain of students’ learning which is of prime importance for their academic success (Bolkan, 2015) also needs to be considered by instructors (Bolkan and Goodboy, 2015). The affective aspect of students’ learning includes their beliefs and attitudes toward “*the course content*,” “*behaviors recommended*,” and “*course instructor*” (McCroskey et al., 1985). As Pogue and AhYun (2006) noted, students’ affective learning also deals with the likelihood of their participation in the course activities and the probability of enrolling in another course with their current instructor. In his study, Wang (2021, p. 2) also postulated that student affective learning pertains to the “outlook and emotional state of students toward the course and the course instructor.” According to Goodboy and Myers (2008), students’ affective learning can be greatly influenced by their instructors’ confirmation. To them, instructors who offer confirmation to their students can improve their affective learning to a large extent. Baker (2010) also stated that students who are instructed by a teacher who employs verbal and nonverbal immediate behaviors in classroom contexts typically demonstrate a higher degree of affective learning. As put forward by Yong (2019), positive teacher–student relationships also affect students’ affective learning in a positive way.

To depict the value of student affective learning, Pekrun et al. (2011) stated that positive affect is the crucial element of educational contexts and is linked with a range of significant outcomes, including students’ internal motivation, self-regulation, perseverance, and grade-point averages. More specifically, Goodboy et al. (2015) submitted that EFL students who have positive and favorable attitudes toward their teachers, instructional materials, and classroom environment are more likely to experience L2 success. Accordingly, investigating the associates and determinants of EFL students’ affective learning seems essential. To address this necessity, several scholars have studied ranges of student-related (e.g., Bigdeli, 2010; Gupta and Pandey, 2018; Wang et al., 2021) and teacher-related factors (e.g., Hsu, 2012; Enskat et al., 2017; Wang and Guan, 2020; Wang, 2021) in relation to EFL students’ affective learning. Yet, teacher–student rapport and teacher support as two valuable teacher-related factors have received limited attention (Federici and Skaalvik, 2014; Yong, 2019). That is, the extent to which EFL students’ affective learning can be predicted by teacher–student rapport and teacher support has remained elusive. To address this gap, this inquiry aims to delve into the impact of teacher–student rapport and teacher support on Chinese EFL students’ affective learning.

Teacher–student rapport, as a potential antecedent of student affective learning, pertains to “a harmonious teacher–student relationship which identified with enjoyment, connection, respect, and mutual trust” (Delos Reyes and Torio, 2021, p. 472). As Falsario et al. (2014) mentioned, through establishing a close and harmonious relationship with pupils, teachers can provide a lively learning atmosphere wherein pupils can gain higher learning outcomes. Bouras and Keskes (2014) also delineated that a strong rapport between students and instructors provides students with an enjoyable learning experience that strengthens their motivation to learn.

Another teacher-related factor that may predict student affective learning is teacher support that refers to “the extent to which students believe their teachers value and seek to establish personal relationships with them” (Chong et al., 2018, p. 3). According to Mercer et al. (2011), those students who perceive their instructors as supportive and helpful tend to put much more effort into classroom activities. This, in turn, leads students toward academic growth and higher learning outcomes (Weyns et al., 2018).

Owing to the importance of teacher support and teacher–student rapport in instructional-learning contexts (Mercer et al., 2011; Bouras and Keskes, 2014), remarkable attention has been devoted to these constructs and their educational consequences (e.g., Feng et al., 2019; Snijders et al., 2020; Noble et al., 2021, to cite a few). Nonetheless, the potential consequences of these constructs for student affective learning have remained elusive. It means that only a few scholars have inspected the capability of teacher support and teacher–student rapport in predicting student affective learning (Federici and Skaalvik, 2014; Yong, 2019). Additionally, to the best of the authors’ knowledge, no inquiry has studied these two teacher-related factors simultaneously to examine their potency in predicting student affective learning. To eliminate these lacunas, the current investigation sought to inspect the impact of teacher support and teacher–student rapport as predictors of Chinese students’ affective learning in English language classes.

## Literature Review

### Teacher–Student Rapport

The term rapport, in a general sense, refers to “an overall feeling between two people encompassing a mutual, trusting, and prosocial bond” (Frisby and Martin, 2010, p. 147). In the educational realm, this concept pertains to a sensitive, warm, and close teacher–student relationship that relies on mutual trust (Roorda et al., 2011). Teachers can build a strong rapport with their pupils by calling them by their first names (Wilson and Ryan, 2013), using humor (Estepp and Roberts, 2015), respecting their ideas (Thompson, 2018), and valuing their academic efforts (Santana, 2019). As Wilson and Ryan (2013) suggested, a close and strong connection between teachers and students will culminate in desirable educational outcomes. To discover the desirable outcomes of teacher–student rapport, some researchers (e.g., Frisby et al., 2016; Yong, 2019; Snijders et al., 2020; Engels et al., 2021) have empirically studied this concept in relation to a range of student-related (e.g., motivation, affective learning, engagement, autonomy, loyalty, etc.) and teacher-related variables (e.g., organizational commitment, satisfaction, etc.). For one, Yong (2019) examined the association of teacher–student rapport with student affective learning. To this end, 286 Malaysian students were invited to complete two open-ended scales designed to measure teacher–student rapport and student affective learning. The findings of this inquiry revealed a positive and close bond between teacher–student rapport and student affective learning. As another instance, Engels et al. (2021) inspected the impact of teacher–student rapport on students’ classroom engagement and academic achievement. To do this, three valid measures of the variables were given to 5,382 Belgian students. Analyzing students’ answers, the researchers discovered a favorable association between teacher–student rapport, student classroom engagement, and academic achievement.

### Teacher Support

The concept of teacher support generally refers to the degree to which instructors care about their pupils, understand their needs, and assist them in attaining their educational goals (Klem and Connell, 2004). As Skinner et al. (2008) mentioned, teacher support as a multidimensional construct can be divided into three main categories, namely “*support for autonomy*,” “*structure*,” and “*involvement*.” Support for autonomy refers to “teachers’ provision of choice, relevance, or respect to students” (Lei et al., 2018, p. 2). Structure as the second dimension of teacher support deals with the coherence and intelligibility of expectations and contingencies. As the last dimension, involvement includes compassion, warmth, devotion of facilities, and understanding the student (Lei et al., 2018). As previous inquiries demonstrated, teacher support is associated with students’ academic engagement (Sadoughi and Hejazi, 2021), academic motivation (Pitzer and Skinner, 2017), academic emotions (Lei et al., 2018), and affective learning (Federici and Skaalvik, 2014). As an instance, Federici and Skaalvik (2014) scrutinized the association of instrumental and emotional teacher support with student affective learning. To do so, the researchers administered two valid questionnaires to 309 Norwegian students. The participants’ answers demonstrated that both instrumental and emotional teacher support can significantly promote student affective learning. In a similar vein, Sadoughi and Hejazi (2021) have delved into the role teacher support in Iranian EFL students’ level of engagement. In doing so, the questionnaires of academic engagement and teacher support were distributed among 450 Iranian English language learners. With regard to the participants’ answers, they found that students’ engagement in English language classes can be remarkably promoted by teacher support.

### Student Affective Learning

Student affective learning generally deals with students’ perceptions and dispositions toward the learning experience (Witt and Wheeless, 2001). As Pogue and AhYun (2006, p. 333) mentioned, student affective learning refers to “student attitudes toward the course, content, and instructor, as well as student attitudes toward anticipated classroom behaviors.” Bekiari (2012) suggested that the manner in which instructors communicate with their learners can drastically influence their affective learning. In line with this premise, numerous studies have inspected the power of teacher communication behaviors, including immediacy, confirmation, and clarity, in predicting students’ affective learning (e.g., Hsu, 2012; Wang, 2021; Wang et al., 2021). Yet, the impact of teacher support and teacher–student rapport as other prime instances of teacher communication behaviors has been inspected by only a few scholars (e.g., Federici and Skaalvik, 2014; Yong, 2019). Moreover, neither in general education nor in language education, no empirical study has simultaneously inspected the consequences of teacher support and teacher–student rapport for students’ affective learning. Additionally, to the best of the researcher’s knowledge, no investigation has been done into the effects of these two communication behaviors on EFL students’ affective learning. To fill the lacunas, the present study intended to evaluate the role of teacher support and teacher–student rapport in Chinese EFL students’ affective learning. To this end, two important research questions were posed:

1. Are there any significant relationships between teacher–student rapport, teacher support, and Chinese EFL students’ affective learning?
2. Do teacher–student rapport and teacher support significantly predict Chinese EFL students’ affective learning?

## Methodology

### Participants

A total of 497 Chinese EFL students were selected using a convenience sampling strategy. Convenience sampling is a prime instance of “*non-probability sampling method*” through which “subjects are typically selected due to their geographical proximity, availability, and easy accessibility” (Dörnyei and Csizér, 2012, p. 82). The sample included 166 males and 331 females, varying in age from 17 to 47 years old (Mean = 21.21, SD = 2.82). Most of the participants (62.7%) were undergraduates (*N* = 312). The rest (37.3%) were postgraduates, including MA students (*N* = 179) and Ph.D. candidates (*N* = 6). To ensure the study’s trustworthiness, all participants were briefed on how to fill out the questionnaires and were convinced that their viewpoints would be kept private.

### Instruments

#### Professor-Student Rapport Scale

To assess students’ perspectives toward the quality of their relationships with their teachers, the “*Professor-Student Rapport Scale* (P-SRS),” designed by Wilson and Ryan (2013), was employed. The P-SRS involves 34 items, each of which is rated on a 5-point Likert scale. Some examples of P-SRS’s items are as follows: item (5) “*My professor is thoughtful*” and item (11) “*My professor encourages questions and comments from students*.” In the current investigation, the reliability of P-SRS was found to be 0.81.

#### Teacher Support Scale

The “*Teacher Support Scale* (TSS)” (McWhirter, 1996) was utilized to assess how supportive teachers are in the eyes of Chinese EFL students. TSS is a valid measure of teacher support that encompasses 27 close-ended items. TSS uses a 5-point Likert scale, varying in responses from 1 “Strongly disagree” to 5 “Strongly agree.” The following are some examples of TSS’s items: item (4) “*My English teacher takes the time to help me get better grades*” and item (18) “*My English teacher supports my goals for the future*.” In the present inquiry, a reliability coefficient of 0.70 was found for this measure.

#### Student Affective Learning Scale

Chinese EFL students’ affective learning was measured *via* “*Student Affective Learning Scale* (SALS)” developed by McCroskey et al. (1985). The SALS comprises five components, including “*Attitude toward the course content*” (items 1–4), “*Attitudes toward behaviors recommended in the course*” (items 5–8), “*Attitude about the teacher*” (items 9–12), “*Actual engagement in the behaviors recommended in the course*” (items 13, 14), and “*Likelihood of taking another course with this teacher*” (items 15, 16). The reliability index of SALS for this study was 0.90.

### Procedure

Initially, the consent form was administered to 550 Chinese EFL students *via* WeChat messenger. The valid measures of the variables (i.e., P-SRS, TSS, and SALS) were then shared among students who indicated their consent by completing the given forms. The respondents were provided with a thorough explanation about the completion of questionnaires. All participants submitted their responses within 4 weeks. The gathered responses were preprocessed to recognize and remove the problematic ones. Then, to inspect the association of teacher support and teacher–student rapport with Chinese EFL students’ affective learning, the composite reliability was utilized. Eventually, to examine the impact of teacher support and teacher–student rapport on Chinese EFL students’ affective learning, SEM was run through the *Smart-PLS* (version 3.3.5). In doing so, the indicator repetition approach, which is essential for running higher order models in *PLS-SEM*, was applied (Ringle et al., 2012).

## Results

At the very beginning, to identify the problematic and missing responses, the collected data were subjected to some pre-processes. Fortunately, no missing or questionable response was found in the collected data. Then, the composite reliability, Cronbach *α*, and convergent validity for each construct were measured. The results revealed that the composite reliability and Cronbach *α* for all three constructs (i.e., teacher–student rapport, teacher support, and student affective learning) were greater than 0.7, indicating a high level of reliability (see Tables 1–3).

**Table 1:** Composite reliability, Cronbach *α*, and convergent validity of the teacher–student rapport.

Teacher–student rapport (RLOC1)		Convergent validity			Reliability	
		Outer loading	*t*-values	AVE	Composite reliability	Cronbach’s *α*
	Indicators	>0.708	>2.57	>0.5	>0.7	>0.7
TSR_01	TSR_01	0.714	10.280	0.685	0.897	0.846
TSR_02	TSR_02	0.724	8.335			
TSR_03	TSR_03	0.719	7.534			
TSR_04	TSR_04	0.755	10.162			
TSR_05	TSR_05	0.709	16.676			
TSR_06	TSR_06	0.746	9.252			
TSR_07	TSR_07	0.781	17.999			
TSR_08	TSR_08	0.777	20.317			
TSR_09	TSR_09	0.749	19.040			
TSR_10	TSR_10	0.735	23.275			
TSR_11	TSR_11	0.846	22.365			
TSR_12	TSR_12	0.752	13.023			
TSR_13	TSR_13	0.781	24.129			
TSR_14	TSR_14	0.761	10.821			
TSR_15	TSR_15	0.821	29.375			
TSR_16	TSR_16	0.781	12.711			
TSR_17	TSR_17	0.812	11.570			
TSR_18	TSR_18	0.755	15.737			
TSR_19	TSR_19	0.767	27.998			
TSR_20	TSR_20	0.849	49.191			
TSR_21	TSR_21	0.826	25.195			
TSR_22	TSR_22	0.790	31.490			
TSR_23	TSR_23	0.864	47.268			
TSR_24	TSR_24	0.792	3.874			
TSR_25	TSR_25	0.859	42.164			
TSR_26	TSR_26	0.866	46.382			
TSR_27	TSR_27	0.886	20.725			
TSR_28	TSR_28	0.827	32.747			
TSR_29	TSR_29	0.755	22.890			
TSR_30	TSR_30	0.715	23.757			
TSR_31	TSR_31	0.834	40.297			
TSR_32	TSR_32	0.865	31.256			
TSR_33	TSR_33	0.736	13.456			
TSR_34	TSR_34	0.755	22.890			

**Table 2:** Composite reliability, Cronbach *α*, and convergent validity of the teacher support.

Teacher support (RHOC1)		Convergent validity			Reliability	
		Outer loading	*t*-values	AVE	Composite reliability	Cronbach’s *α*
	Indicators	>0.708	>2.57	>0.5	>0.7	>0.7
Invested (RLOC2)	Invst_01	0.762	19.470	0.731	0.956	0.974
	Invst_02	0.851	30.798			
	Invst_03	0.880	63.977			
	Invst_04	0.917	99.231			
	Invst_05	0.814	37.219			
	Invst_06	0.903	63.360			
	Invst_07	0.916	84.341			
	Invst_08	0.782	28.645			
Emot sup (RLOC3)	Emotsup_01	0.916	82.805	0.828	0.960	0.948
	Emotsup_02	0.895	54.352			
	Emotsup_03	0.900	72.027			
	Emotsup_04	0.933	80.426			
	Emotsup_05	0.905	68.394			
Expect (RLOC4)	Expect_01	0.911	82.044	0.838	0.963	0.952
	Expect_02	0.904	62.985			
	Expect_03	0.923	89.111			
	Expect_04	0.894	55.457			
	Expect_05	0.944	137.848			
Inform sup (RLOC5)	Infrmsup_01	0.898	64.114	0.829	0.936	0.897
	Infrmsup_02	0.940	122.595			
	Infrmsup_03	0.892	48.460			

**Table 3:** Composite reliability, Cronbach *α*, and convergent validity of the student affective learning.

Student affective learning (RHOC2)		Convergent validity			Reliability	
		Outer loading	*t*-values	AVE	Composite reliability	Cronbach’s *α*
	Indicators	>0.708	>2.57	>0.5	>0.7	>0.7
Attitude toward course content (RLOC6)	AttCC_01	0.780	23.623	0.847	0.943	0.909
	AttCC_02	0.789	36.156			
	AttCC_03	0.781	23.363			
	AttCC_04	0.759	25.711			
Attitudes toward behaviors recommended in the course (RLOC7)	AttBRC_01	0.840	36.452	0.834	0.938	0.900
	AttBRC_02	0.821	26.157			
	AttBRC_03	0.782	20.691			
	AttBRC_04	0.846	12.932			
Attitude about the teacher (RLOC8)	AttT_01	0.835	32.925	0.784	0.957	0.946
	AttT_02	0.796	23.142			
	AttT_03	0.835	19.489			
	AttT_04	0.855	25.412			
Actual engagement in the behaviors recommended in the course (RLOC9)	ActuEB_01	0.892	33.492	0.840	0.969	0.962
	ActuEB_02	0.719	28.179			
Likelihood of taking another course with this teacher (RLOC10)	LikTCT_01	0.888	29.145	0.590	0.934	0.920
	LikTCT_02	0.799	26.125			

Then, to assess the discriminant validity of the sub-constructs, the Fornell-Larcker criterion was utilized. The results demonstrated that the square root of average variance extracted (AVE) was higher than the inter-correlations of the sub-constructs (Table 4).

**Table 4:** Discriminant validity of the sub-constructs.

Sub-constructs	Fornell-Larcker criterion									
	ActuEB	AttBRC	AttCC	AttT	Emot sup	Expect	Inform Sup	Invested	LikTCT	TSupprt
ActuEB	0.892									
AttBRC	0.768	0.823								
AttCC	0.731	0.756	0.761							
AttT	0.782	0.789	0.705	0.831						
Emot Sup	−0.55	−0.531	−0.505	−0.577	0.91					
Expect	−0.542	−0.54	−0.517	−0.579	0.902	0.916				
Inform Sup	−0.524	−0.514	−0.509	−0.536	0.865	0.881	0.91			
Invested	−0.529	−0.522	−0.514	−0.55	0.819	0.895	0.849	0.855		
LikTCT	0.714	0.647	0.62	0.669	−0.512	−0.516	−0.501	−0.501	0.886	
TSupprt	−0.558	−0.549	−0.532	−0.585	0.873	0.867	0.718	0.568	−0.528	0.857

Furthermore, the correlations between the three constructs were inspected. The results evinced that teacher–student rapport was strongly correlated with student affective learning (*r* = 0.436). Similarly, teacher support was found to be significantly correlated with student affective learning (*r* = 0.436). A weak correlation was also found between teacher–student rapport and teacher support (*r* = 0.128).

**Figure 1:**
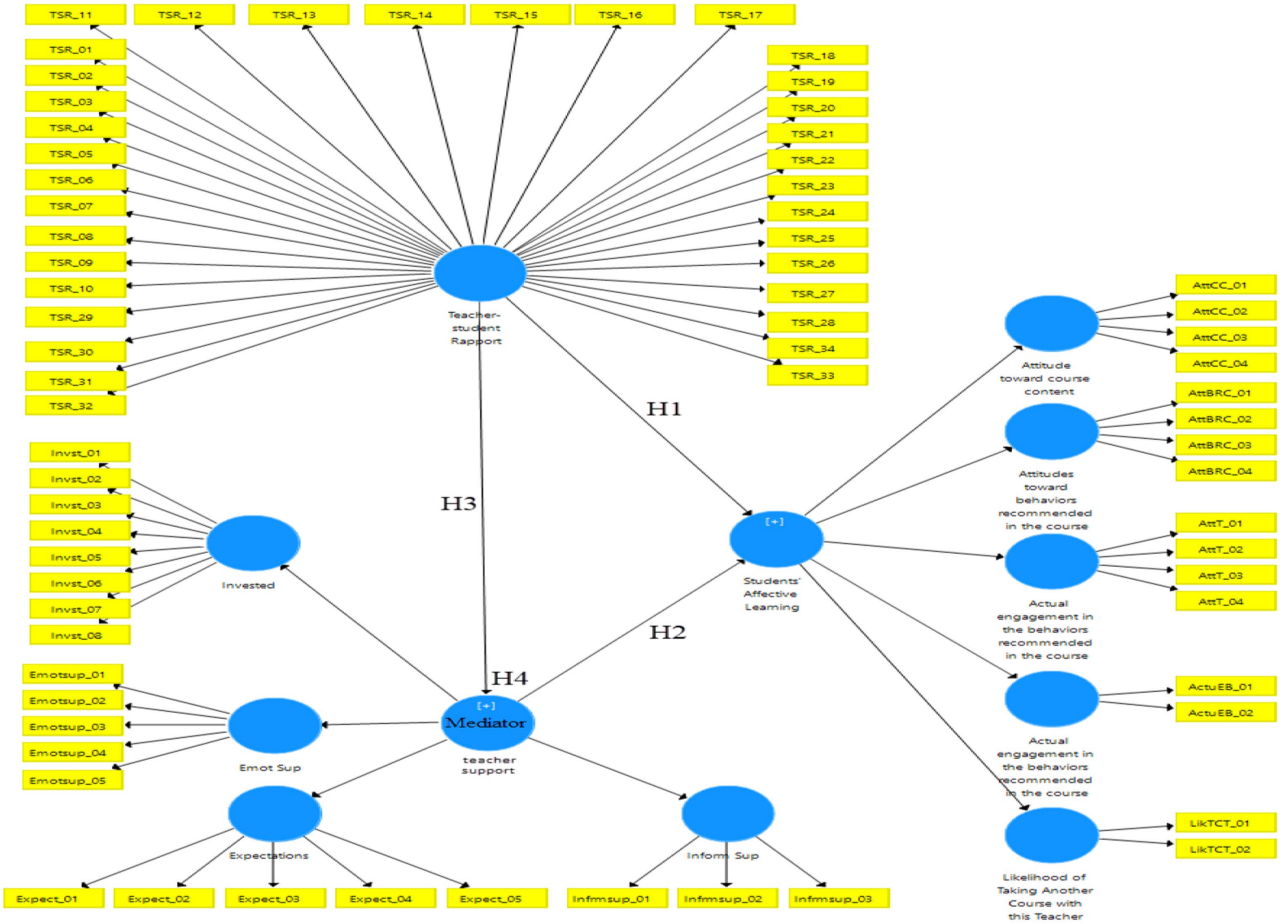
The structural model of the associations between teacher–student rapport, teacher support, and student affective learning.

Finally, to delve into the role of teacher support and teacher–student rapport as predictors of Chinese EFL students’ affective learning, SEM was performed using the Smart-PLS software. Figure 1 depicts the structural model of associations between teacher–student rapport, teacher support, and student affective learning.

To test the structural model of the associations between teacher–student rapport, teacher support, and student affective learning bootstrapping was performed *via* the Smart-PLS software. The results were thoroughly presented in Table 5.

**Table 5:** The results of testing the structural model.

IDs	Hypotheses	Standardized coefficient (*β*)	*t*-value	*f*2	*R* ^2^
H_1_	Do teacher–student rapport significantly predict Chinese EFL students’ affective learning?	0.176^***^	3.624	0.048	0.436
H_2_	Do teacher support significantly predict Chinese EFL students’ affective learning?	0.576^***^	14.315	0.513	0.436
H_3_	Do teacher–student rapport significantly predict teacher support?	0.358^***^	7.513	0.147	0.128
H_4_	Do teacher support mediates the relationship between teacher-student rapport and students’ affective learning?	0.206^***^	7.322	NA	_

*** *It shows significance*.

As shown in Table 5, to determine how much of the variation in Chinese EFL students’ affective learning could be attributed to teacher–student rapport and teacher support, the standardized estimates were calculated. Accordingly, both teacher–student rapport (*β* = 0.176, *t* = 3.624, *p* < 0.001) and teacher support (*β* = 0.576, *t* = 14.315, *p* < 0.001) were found to be strong antecedents of Chinese EFL students’ affective learning.

## Discussion

The present article was primarily set out to examine the interrelationships between Chinese EFL students’ affective learning, teacher–student rapport, and teacher support. Correlational analyses revealed strong and positive associations, first, between teacher–student rapport and student affective learning, and second, between teacher support and student affective learning. Concerning the positive association between teacher–student rapport and student affective learning, it can be mentioned that this result appears to be in line with that of Yong (2019), who found a positive and close bond between teacher–student rapport and Malaysian students’ affective learning. It is also encouraging to compare this result with that discovered by Engels et al. (2021) who found a favorable correlation between teacher–student rapport and students’ learning outcomes. Besides, the discovered relationship between teacher support and student affective learning accords with findings of Federici and Skaalvik (2014), which demonstrated that a remarkable association exists between instrumental and emotional teacher support and students’ affective learning.

Additionally, this study also aimed to inspect the role of teacher–student rapport and teacher support as predictors of Chinese EFL students’ affective learning. Put simply, the current inquiry was intended to find out how much of the variation in Chinese EFL students’ affective learning may be attributed to teacher–student rapport and teacher support. As the structural model indicated, teacher–student rapport was found to be a strong antecedent of Chinese EFL students’ affective learning. That is, a strong and friendly relationship between teachers and pupils can impact students’ affective learning. This result resonates with that of Snijders et al.’s (2020) study, highlighting the favorable influence of student-faculty relationships on student learning outcomes. Besides teacher–student rapport, teacher support had a favorable influence on Chinese EFL students’ affective learning, as represented by the structural model. This supports the ideas of Mercer et al. (2011) and Wang and Guan (2020) also asserted that supportive instructors can largely influence their students’ learning outcomes, including affective learning.

## Conclusion

The present investigation attempted to delve into the function of teacher support and teacher–student rapport in predicting Chinese EFL students’ affective learning. The results of correlational analyses and structural equation modeling uncovered that teacher–student rapport and teacher support serve a facilitative function in raising Chinese students’ affective learning outcomes. Put simply, teacher–student rapport and teacher support can positively affect Chinese students’ affective learning. Therefore, it could conceivably be concluded that those EFL students who enjoy a favorable relationship with their teachers and receive constant support and assistance are more likely to attain high learning outcomes. This appears to be highly beneficial and illuminating for EFL teachers and teacher educators. To enhance EFL students’ affective learning outcomes, teachers should establish a close bond with their pupils. They are also required to support students in different stages of language learning. In this regard, teacher educators are expected to instruct EFL teachers on how to build strong relationships with students. They are also required to train EFL teachers to be supportive in instructional-learning contexts.

Finally, some limitations need to be mentioned concerning the current study. First, a quantitative method was adopted to conduct this investigation. Future studies are recommended to use a mixed-method approach to come up with more comprehensive results. Second, in this study, only close-ended questionnaires were used to gather the required data. Further research should therefore employ other data collection instruments (e.g., open-ended questionnaires, structured/semi-structured interviews, etc.) to triangulate data. Third, the mediating effect of contextual variables such as gender, age, and educational background was overlooked, which should be examined in future research.

## Data Availability Statement

The original contributions presented in the study are included in the article/supplementary material, further inquiries can be directed to the corresponding author.

## Ethics Statement

The studies involving human participants were reviewed and approved by China University of Petroleum (East China) Academic Ethics Committee. The patients/participants provided their written informed consent to participate in this study.

## Author Contributions

All authors listed have made a substantial, direct, and intellectual contribution to the work and approved it for publication.

## Funding

The study was supported by “the Fundamental Research Funds for the Central Universities (Grant No. 17CX04042B)”; “the Teaching Research and Reform Project of China University of Petroleum (East China) (Grant No. KC-202063)”; and “the Education and Science Planning Project of Shandong Province in China (Grant No. 2021WYB014)”.

## Conflict of Interest

The authors declare that the research was conducted in the absence of any commercial or financial relationships that could be construed as a potential conflict of interest.

## Publisher’s Note

All claims expressed in this article are solely those of the authors and do not necessarily represent those of their affiliated organizations, or those of the publisher, the editors and the reviewers. Any product that may be evaluated in this article, or claim that may be made by its manufacturer, is not guaranteed or endorsed by the publisher.
